# High-Tech Acupuncture and Integrative Laser Medicine 2014

**DOI:** 10.1155/2015/878620

**Published:** 2015-08-04

**Authors:** Gerhard Litscher, Xin-Yan Gao, Lu Wang, Bing Zhu

**Affiliations:** ^1^Research Unit for Complementary and Integrative Laser Medicine, Research Unit of Biomedical Engineering in Anesthesia and Intensive Care Medicine, TCM Research Center Graz, Medical University of Graz, 8036 Graz, Austria; ^2^Institute of Acupuncture and Moxibustion, China Academy of Chinese Medical Sciences, Beijing 100700, China

The special issue focuses on the latest innovative aspects of high-tech acupuncture, including related research topics. In this year, the accepted papers deal with the following interesting aspects.

L. Zhang et al. investigated the inhibiting effect of electroacupuncture (EA) at Zusanli on early inflammatory factor levels formed by postoperative abdominal adhesions in a rat model, to find out the relationship between EA and the cholinergic anti-inflammatory pathway. Sixty-four male Wistar rats were divided into 8 groups. The *α*-bungarotoxin (*α*-BGT) was injected into the abdominal cavity after surgery, and the bilateral celiac vagotomy was done during the surgery. Three days later, the levels of inflammatory mediators in tissues were evaluated. The abdominal adhesion groups developed obvious edema. It was found that EA lowered the elevated levels of inflammatory mediators significantly; EA plus *α*-BGT and vagotomy showed less anti-inflammatory effects. The authors concluded that the activation of the cholinergic anti-inflammatory pathway might be one of the mechanisms of EA at Zusanli points to exert the anti-inflammatory effects.

Effects of Guasha on heart rate variability (HRV) were investigated by X. Wang et al. in ten healthy male volunteers and 15 weightlifters. These test persons were divided into two groups and underwent 20-minute Guasha therapy. Electrocardiography was recorded before and immediately after Guasha. HRV was calculated in both the time domain and frequency domain. The different evaluated HRV parameters reacted differently to the Guasha therapy. In conclusion, Guasha therapy facilitates the parasympathetic nervous activity and modulates the balance between parasympathetic and sympathetic activities in both healthy men under normal conditions and weightlifters after training sessions. Although the changes of the HRV parameters were similar in both groups, the responsiveness was more pronounced in healthy men than in male weightlifters.

İ. Öztekin et al. conducted a study aimed to compare anti-inflammatory effects of oligonol, acupuncture, and quantum light therapy in rat models of estrogen-induced prostatitis. Ninety adult male Wistar albino rats were divided into nine different experimental groups. Chronic prostatitis (CP) was induced by the administration of 17-beta-estradiol (E2) and dihydrotestosterone. Oligonol was given for 6 weeks. Acupuncture needles were inserted at CV 3/4 and bilaterally B 32/35 points. Quantum therapy was administered three times weekly for 6 weeks. Lateral lobes of prostates were dissected for histopathologic evaluation. All treatment modalities tested in this study showed anti-inflammatory effects in the treatment of CP. The authors found a synergistic effect for oligonol plus quantum light combination, whereas monotherapy with oligonol showed a superior anti-inflammatory efficacy as compared to quantum light and acupuncture monotherapies.

J. Wu et al. performed a systematic review of adverse effects of acupuncture in China as a further step towards the modernization of acupuncture. This review took note of the frequency and severity of adverse complications and events in acupuncture treatment reported in China from 1980 to 2013. Over the 33 years, 182 incidents were identified in 133 relevant papers. Internal organ, tissue, or nerve injury was found to be the main complications of acupuncture especially for pneumothorax and central nervous system injury. Adverse effects also included syncope, infections, hemorrhage, allergy, burn, aphonia, hysteria, cough, thirst, fever, somnolence, and broken needles. Therefore, the authors concluded that qualifying training of acupuncturists should be systemized and the clinical acupuncture operations should be standardized in order to effectively prevent the occurrence of acupuncture accidents.

A literature review and analysis of neuroimaging and neuromonitoring effects of electro- and manual acupuncture on the central nervous system was performed by B. E. Scheffold et al. The PubMed Database was searched from 1/1/2000 until 1/6/2014 with restriction on human studies in English language. Only studies comparing manual or electroacupuncture with sham acupuncture were eligible. In 29 out of 33 studies, verum acupuncture results were found to present either more or different modulation effects on neurological components measured by neuroimaging and neuromonitoring methods than sham acupuncture. Only four studies reported no effects of verum in comparison to sham acupuncture. Evaluation of the very heterogeneous results showed evidence that verum acupuncture elicits more modulation effects on neurological components than sham acupuncture.

A pilot study by T. K. Leung et al. investigated the effects of bioceramic (BC) irradiation and resonant sound waves on meridian channels. BC is a material which emits nonionizing radiation and luminescence induced by visible light. It also facilitates the break-up of large clusters of water molecules by weakening hydrogen bonds, which is one of the key mechanisms underlying the effects of BC. In their study, the authors used sound to amplify the effect of BC. Thirteen patients severely affected in their sleep patterns and life quality were enrolled in a trial of BC resonance (i.e., rhythmic 100-dB sound waves with frequency set at 10 Hz) applied to the skin surface of the anterior chest. According to the preliminary data, a “propagated sensation along meridians” was experienced in all BC resonance-treated patients but not in any of the nine control patients. The authors believe that the device enhances microcirculation through a series of biomolecular and physiological processes and subjects the specific meridian channels of traditional Chinese medicine to coherent vibration. They conclude that this noninvasive technique may offer an alternative to needle acupuncture.

B. Jia et al. investigated the effects of acupuncture at real or sham acupoints on the intrinsic brain activity in mild cognitive impairment (MCI, a transitional phase between normal function and Alzheimer disease (AD)) patients. Previous studies established that acupuncture at different acupoints could exert different modulatory effects on the brain network. However, based on the pathology characteristics of MCI and AD, whether acupuncture at real or sham acupoints can produce different effects on the brain network in MCI or AD patients remains unclear. The authors used resting state fMRI and reported that acupuncture at Taixi (KI3) induced amplitude of low frequency fluctuation (ALFF) change of different brain regions in MCI patients from those shown in the healthy controls. In resting state, acupuncture at the sham acupoint in MCI patients activated brain regions different from those in healthy controls. Therefore, the authors concluded that, in MCI patients, acupuncture at KI3 and sham acupoint improved the neuronal activities of certain cognitive-related regions.

The objective of the study by A. Santoro et al. was to investigate if auriculotherapy (AT) can modulate pain threshold. Two groups of healthy volunteers were enrolled in the study. AT consisted of placing vaccaria seeds over the “fingers point” of one ear, and sham treatment consisted of a puncture/massage above the skin of the neck. Each subject was asked to perform an autoalgometric test on three occasions (before, 1 hour after, and 24 hours after AT), until a minimum pain sensation or a maximally tolerable pain sensation. The results showed a significantly higher pain threshold in the maximal test at 24 hours after AT compared to sham treatment. This result indicated for the first time that AT can increase pain tolerability, rather than affecting the minimal pain threshold.

J. H. Cho et al. explored effects of Jae-Seng acupuncture treatment, a newly attempted bloodletting therapy, on the improvement of nasolabial folds and eye wrinkles. The microneedle therapy system, a mechanical method involving making multiple minute holes in the skin, reportedly improves skin condition. According to the authors, Jae-Seng acupuncture has several advantages over traditional mechanical punching methods because it allows the practitioner to regulate the depth and direction of needle stimulations. The nasolabial folds and eye wrinkles of 107 patients were subjected to a digital skin image analyzer, before the treatment and one month after treatment. Additionally, stimulation of some meridians was performed to improve the function of these vessels. Analyses of the images indicated that Jae-Seng acupuncture improved eye wrinkles, suggesting that this technique is a safe and effective method for the improvement of facial skin conditions.

Within a pilot study, T. Huang applied electrothermal Bian-stone stimulation to Guangming (GB37) to relieve asthenopia in 15 female patients. The results of this controlled pilot study showed significant increases in the eyes' temperature. At the same time, no changes were found following stimulation at the control points. Furthermore, after warm stimulation on Guangming, the clinical symptoms were getting better than using the control points. The symptoms score also decreased significantly. The author states that it was demonstrated that there is some relationship between the Guangming (GB37) acupoint and the eyes and that warm stimulation on Guangming could relieve the uncomfortableness of asthenopia.

S. Ono and Y. Mukaino tested the efficacy and cost effectiveness of an acupuncture treatment using a new skin stimulus tool called* M*-Test which is a measure based on symptoms accompanied with body movements within a pragmatic randomized controlled trial targeting hemodialysis patients. The authors stated that* M*-Test can simultaneously reduce hemodialysis patients' diverse symptoms. Its diagnosis and treatment are based on simple movements that can be performed by anyone and allow determining which meridians have problems by analyzing symptoms accompanied with movement. It also enables conducting a safe and effective treatment with use of microcorn which is a noninvasive treatment tool. This time they conducted microcorn intervention on hemodialysis patients based on the diagnosis of* M*-Test. As a result, almost all of dialysis patients' complaints were relieved while the score of health-related quality of life increased. According to the authors' calculation of cost effectiveness, it was confirmed to be very cost-effective.

